# Cardiac Radiographic Measurements in Ferrets Using the OsiriX MD Programme

**DOI:** 10.3389/fvets.2021.795947

**Published:** 2022-01-10

**Authors:** Alejandro Gutiérrez, Luis J. Ezquerra, Pedro L. Rodríguez, Joaquín Jiménez

**Affiliations:** ^1^Departamento de Medicina Animal, Facultad de Veterinaria, Universidad de Extremadura, Caceres, Spain; ^2^Departamento de Produccion Animal y Ciencia de los Alimentos, Universidad de Extremadura, Caceres, Spain

**Keywords:** ferret, X-ray, measurement, vertebral heart scale, sternebral heart scale, OsiriX

## Abstract

**Objectives:** To adapt the vertebral heart scale (VHS) for use in ferrets and identify new scales and tools that allow to establish the normal heart size by means of radiography more quickly and effectively.

**Methods:** Forty healthy pet ferrets (*Mustela putorius furo*) were used in this prospective study. The measurements were made on right lateral, left lateral, ventrodorsal, and dorsoventral projections, using OsiriX MD medical imaging software, to evaluate sex effect and variance within the different heart scales. Cardiac measurements were also correlated to VHS and the cardiac dimension in the same projection.

**Results:** Most of the cardiac measurements were significantly different between males and females. The results for the VHS were: right lateral VHS (RL-VHS): 5.52 ± 0.28 v (vertebrae units); left lateral (LL-VHS): 5.55 ± 0.28 v; and dorsoventral VHS (DV-VHS): 6.22 ± 0.34 v for males and RL-VHS: 5.24 ± 0.2 v; LL-VHS: 5.25 ± 0.20 v; and DV-VHS: 5.97 ± 0.35 v for females. Regarding the sternebral heart scale (SHS), the values were: RL-SHS: 5.10 ± 0.20 s (sternebrae units) and LL-SHS: 5.11 ± 0.20 s for males and RL-SHS: 4.67 ± 0.24 s and LL-SHS: 4.67 ± 0.28 s for females. The new measurements based on determining the cardiac area were also marked by clear sexual dimorphism, as shown for the cardiac area-axis (AREA-AXIS): RL-AREA-AXIS: 3.82 ± 0.45 cm^2^; LL-AREA-AXIS: 3.87 ± 0.41 cm^2^; ventrodorsal (VD)-AREA-AXIS: 4.59 ± 0.64 cm^2^; and DV-AREA-AXIS: 4.80 ± 0.50 cm^2^ for males and RL-AREA-AXIS: 2.39 ± 0.23 cm^2^; LL-AREA-AXIS: 2.41 ± 0.26 cm^2^; VD-AREA-AXIS: 3.08 ± 0.45 cm^2^; and DV-AREA-AXIS: 3.06 ± 0.47 cm^2^ for females. The cardiac area open polygon (AREA-POL) values were: RL-AREA-POL: 6.78 ± 0.65 cm^2^; LL-AREA-POL: 6.88 ± 0.68 cm^2^; VD-AREA-POL: 7.20 ± 0.91 cm^2^; and DV-AREA-POL: 7.57 ± 0.88 cm^2^ for males and RL-AREA-POL: 4.28 ± 0.30 cm^2^; LL-AREA-POL: 4.35 ± 0.35 cm^2^; VD-AREA-POL: 4.72 ± 0.65 cm^2^; and DV-AREA-POL: 4.79 ± 0.66 cm^2^ for females, with similar differences noted from various radiographic projections. A good correlation was noted between VHS and SHS, and a very strongly positive correlation existed between cardiac area measurements and cardiac dimensions.

**Conclusion:** The VHS adapted to ferrets, the SHS, as well as the cardiac area measurements presented in our study are ideal tools for the assessment of cardiac size in ferrets.

## Introduction

Heart disease has already been described in ferrets, with dilated cardiomyopathy being the most common pathology (1, 2). Radiographic evaluation of cardiac dimensions may be important for the initial evaluation of heart disease, as it may reveal altered anatomical structures secondary to eccentric enlargement.

Currently, the most objective radiographic method for the quantitative diagnosis of heart disease in different animal species is the vertebral heart scale (VHS), which relates the size of the heart to the length of the body without considering the shape of the thorax (3). Lately, this vertebral measurement system of the heart size has been used by other authors in different animal species (4–9) and in different breeds (10–17), suggesting specific values for the vertebral heart size. Although earlier publications described standardised measurements of heart size in ferrets, Stepien et al. (18) proposed a study to establish a range of normal values for cardiac dimensions; the scale created was the VHS with two modifications. The first modification is that the long and short axes are compared with vertebral lengths starting at the cranial edge of T5 and estimated at 0.25 vertebrae (instead of 0.1 vertebrae). The second modification is that the measurements of the ventrodorsal (VD) projection are compared with the vertebral lengths of the same view. In the original study (3), the measurements of the cardiac silhouette in the VD projection were compared with the vertebral measurements obtained in the right lateral (RL) projection (18). Later, in 2009, another study was published in which the authors described a different VHS (18); the values obtained from the cardiac long axis (LA) and short axis (SA) measurements were combined and then correlated to the length obtained by counting the thoracic vertebrae from the cephalic side of the sixth thoracic vertebra. This scale was also used to evaluate sex and weight differences (19).

The VHS method is fairly reliable for evaluating generalised cardiomegaly and some types of cavity enlargement such as enlargement of the left atrium. It has several disadvantages in assessing size changes of the right atrium and ventricular enlargement (3). In addition, in the presence of congenital abnormalities of the ferret spine (20), the VHS values may be erroneously high, leading to a false diagnosis of cardiomegaly.

Digital radiography and medical imaging workstations are replacing conventional radiography in both human and veterinary medicine and research (21). OsiriX MD, a software for Mac platform, is one of the most widely used software programs for the description and validation of new scientific research methods due to its flexibility, accuracy, and reliability (22–24).

Therefore, the objectives of this study were (1) to adapt the VHS—established in other animal species—for use in ferrets, considering sex and the different radiographic projections, (2) to determine its correlation with the new sternebral heart scale (SHS) based on the normalised measurements of the long and short axes of the heart, and (3) to describe new radiographic measurements to assess cardiac size in ferrets. It was also hypothesised that the OsiriX MD medical imaging software will allow the acquisition of new scales and tools to determine the normal heart size of ferrets by using X-rays.

## Materials and Methods

For this prospective study, 40 healthy, non-castrated pet ferrets (*Mustela putorius furo*) were used. All 40 ferrets were subjects of the Clinical Veterinary Hospital of the University of Extremadura. They had no cardiorespiratory pathologies, as confirmed by physical examination, auscultation, and echocardiography, according to the reference values (25, 26). An additional inclusion criterion was a minimum age of 6 months, since this is the age at which they reach their adult size and sexual maturity (27). Subjects who, after cardiac evaluation, suffered from some pathological process that could influence their cardiovascular status were excluded from the study.

In all ferrets, echocardiography and chest X-ray were performed under sedation [butorphanol (Torbugesic ® Vet, Zoetis, Madrid, Spain, 0.2 mg/kg), butorphanol-acepromazine (Torbugesic® Vet, Zoetis, Madrid, Spain, 0.2 mg/kg; Calmivet ®, Vetoquinol, Madrid, Spain, 0.1 mg/kg) or acepromazine-buprenorphine (Calmivet ®, Vetoquinol, Madrid, Spain, 0.1 mg/kg; Buprecare ®, Ecuphar NV, Oostkamp, Belgium, 0.01 mg/kg)], due to the low cardiovascular effects. Data such as age, sex, and body weight were routinely collected.

### Radiographic Measurements

For each ferret, radiographic measurements were made in the RL, left lateral (LL), ventrodorsal (VD), and dorsoventral (DV) projections. The X-ray device used was Philips (OP80) with image acquisition at 66 kVp and 6 mAs, and a focal-film distance of 1 m. All scanned X-rays were performed using the Kodak DirectView CR500 Cassette and processed in Kodak DirectView CR500 System software version 4.5, Veterinary software 2.1. The images were analysed by the same researcher and processed using OsiriX MD medical imaging software. Only good-quality X-rays were used to obtain measurements. The measurements obtained in each projection were developed using the ROIs (length, axis, and open polygon) tools of the software.

**Figure 1:**
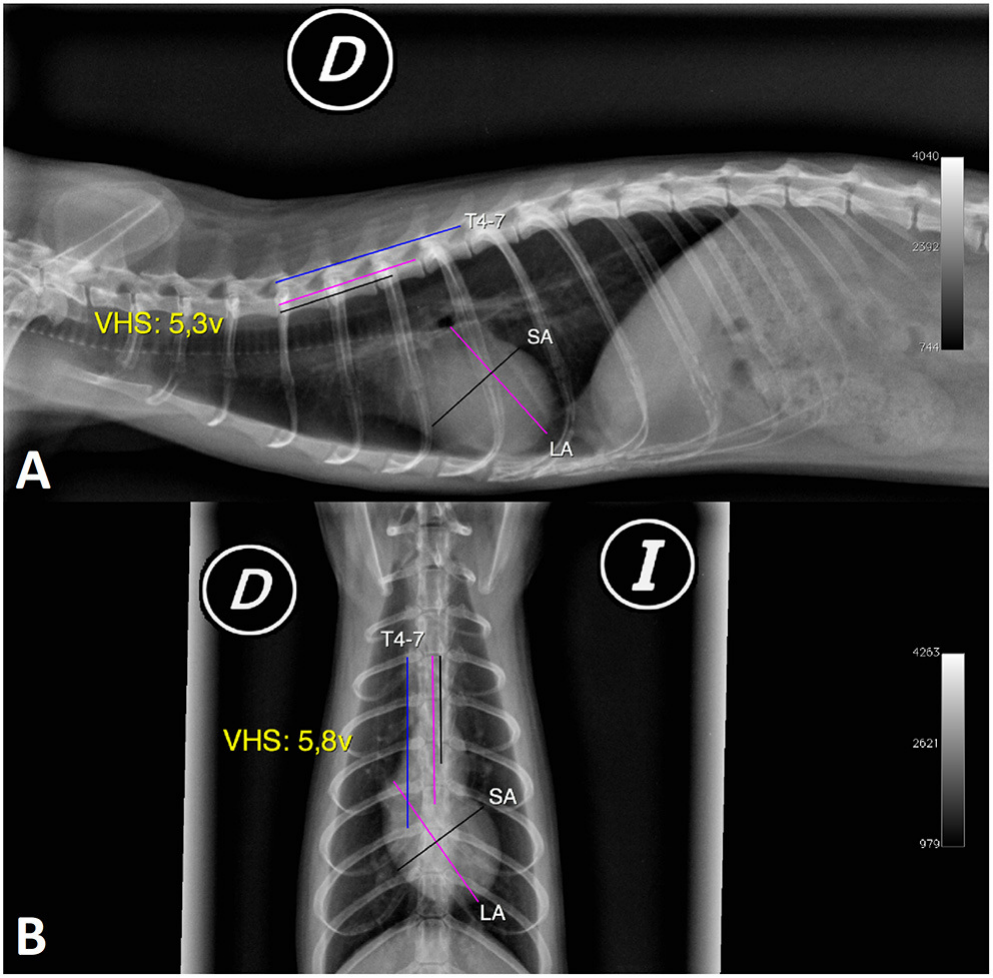
Radiographic image in the right lateral (RL) and ventrodorsal (VD) projections of the normal thorax of a female ferret **(A,B)**. X-rays show measurements of the cardiac silhouette. Long axis of the cardiac silhouette (LA), short axis of the cardiac silhouette (SA), vertebral body length from T4 to T7 (T4–7), and vertebral heart scale (VHS) (D, right; I, left).

**Figure 2:**
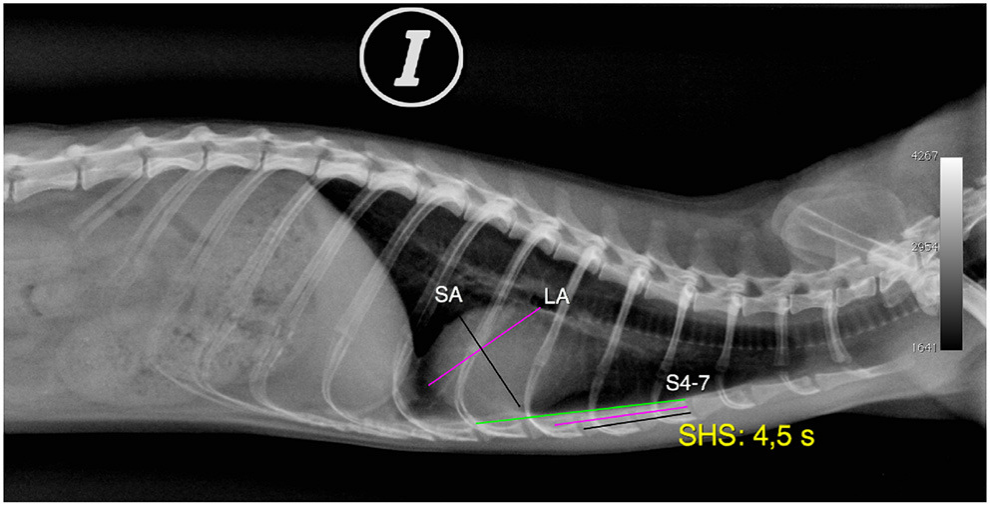
Radiographic image in the left lateral (LL) projection of the normal thorax of a female ferret. X-rays show measurements of the cardiac silhouette. Long axis of the cardiac silhouette (LA), short axis of the cardiac silhouette (SA), sternebral body length from S4 to S7 (S4–7), and sternebral heart scale (SHS) (I, left).

The long axis of the cardiac silhouette in the right lateral and left lateral projections (RL-LA, LL-LA) was defined as the length of the heart from the ventral edge of the tracheal bifurcation (carina) to the apex of the heart in the right and left lateral projections expressed in centimetres. This dimension reflects the combined size of the atrium and left ventricle (Figures 1A, 2).

The short axis of the cardiac silhouette in the right and left lateral projections (RL-SA, LL-SA) was defined as the maximum width of the heart measured in the middle third, perpendicular to the measurement of the LA of the cardiac silhouette in the right and left lateral projections, expressed in centimetres. This measurement includes the left and right chambers of the heart in the region of the atrioventricular sulcus (Figures 1A, 2).

The long axis of the cardiac silhouette in the ventrodorsal and dorsoventral projections (VD-LA, DV-LA) was defined as the length of the heart measured from the middle of the cranial edge of the cardiac silhouette to the apex in the ventrodorsal and dorsoventral projections, expressed in centimetres (Figure 1B).

The short axis of the cardiac silhouette in the ventrodorsal and dorsoventral projection (VD-SA, DV-SA) was defined as the width of the heart measured in the middle third, perpendicular to the LA of the cardiac silhouette in the ventrodorsal and dorsoventral projections, expressed in centimetres (Figure 1B).

The sum of L + S in the right and left lateral projections (RL-L + S, LL-L + S) was defined as the sum of the long and short axes in the right and left lateral projections, expressed in centimetres.

The sum of L + S in the ventrodorsal and dorsoventral projections (VD-L + S, DV-L + S) was defined as the sum of the long and short axes in the ventrodorsal and dorsoventral projections, expressed in centimetres.

The length of T4–7 in the right and left lateral views (RL-T4–7, LL-T4–7) was defined as the total length of the four vertebrae measured from the cranial edge of T4 to the caudal edge of T7 in the right and left lateral projections, expressed in centimetres (Figure 1A).

The length of T4–7 in the ventrodorsal and dorsoventral projections (VD-T4–7, DV-T4–7) was the length of the vertebral body of T4–7 measured in the VD and DV radiographic views, in the same way as mentioned for the lateral view and expressed in centimetres (Figure 1B).

Regarding the VHS in right lateral, left lateral, ventrodorsal, and dorsoventral projections (RL-VHS, LL-VHS, VD-VHS, and DV-VHS), the measurements of the long and the short axes were done along the spine, starting at the cranial edge of the fourth thoracic vertebra (T4). The size of the heart, expressed as the number of vertebrae, was estimated to have an accuracy of 0.1 vertebrae. The sum of the SA and LA, expressed in vertebral units (v), determined the VHS in each projection. This measure was based on the guidelines originally published by Buchanan and Bücheler in dogs (3) (Figures 1A,B).

The length of 4–7 sternebrae in the right and left lateral views (RL-S4–7, LL-S4–7) was defined as the total length of four sternebrae measured from the cranial edge of the fourth sternebra to the caudal edge of the seventh sternebrae in the right and left lateral projections, expressed in centimetres (Figure 2).

Regarding the SHS in right and left lateral projections (RL-SHS, LL-SHS), the long and the short axes were measured along the sternum starting at the cranial edge of the fourth sternebra (S4). The size of the heart, expressed as the number of sternebrae, is estimated to have an accuracy of 0.1 sternebrae. The sum of the SA and LA expressed in sternebral units determines the SHS in each projection. The measurement is expressed in sternebral units (s). Furthermore, if the measurement of the short and the long axes, expressed in sternebrae, is in the centre of the sternocostal joint, the measurement is made to the anterior sternebra. However, if contact is made with the cranial edge of the next sternebra, 0.1 is added to the sternebral value (Figure 2).

**Figure 3:**
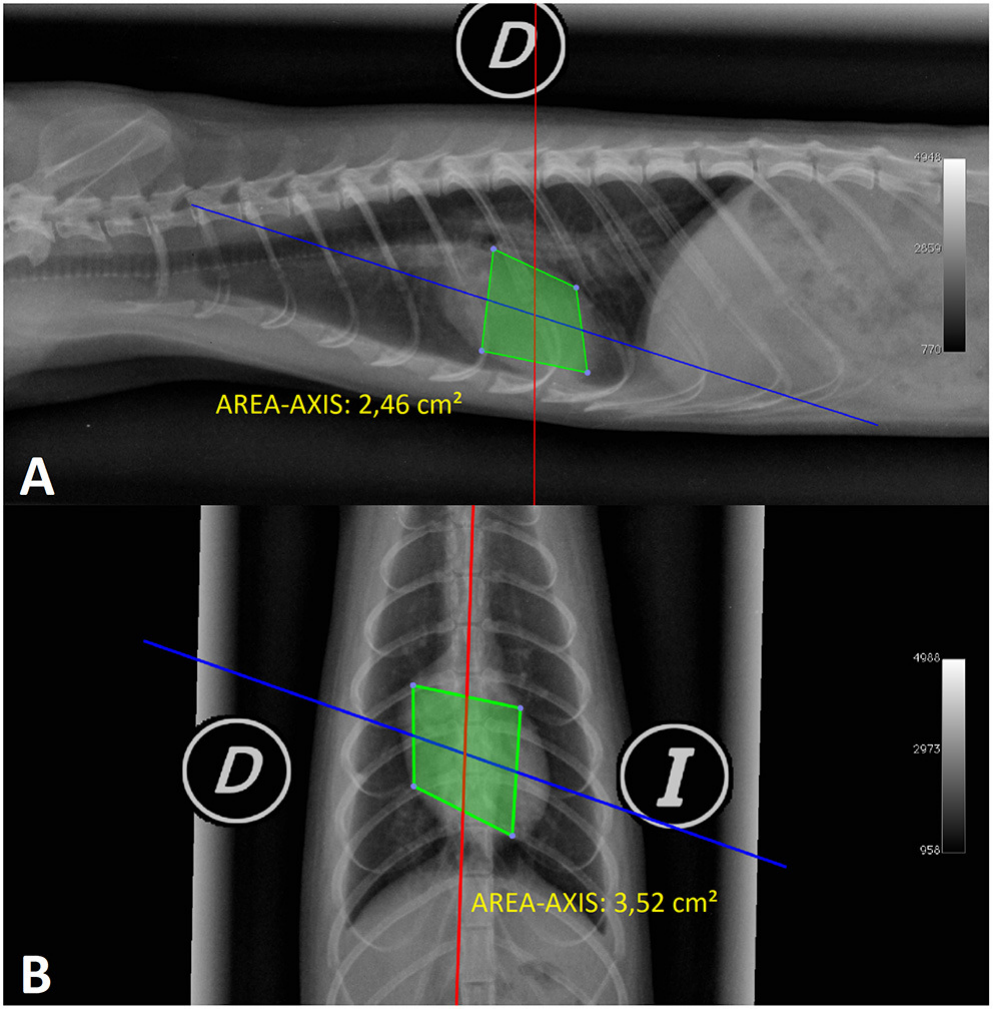
Radiographic image in the right lateral (RL) and ventrodorsal (VD) projections of the normal thorax of a female ferret **(A,B)**. The X-rays show a measure of axis cardiac area (AREA-AXIS) in both projections (D, right; I, left).

The axis cardiac area measurement in the right and left lateral projections (RL-AREA-AXIS, LL-AREA-AXIS) was created using the OsiriX software axis region of interest (ROI). For this purpose, four points were placed as if they were the LA and the SA of the cardiac silhouette as follows: the first point was placed on the ventral edge of the tracheal bifurcation; the second point was placed in the middle third, perpendicular to the imaginary LA of the cardiac silhouette starting from the right side of the heart; the third point was placed at the apex of the heart to form the imaginary axis (LA); and the fourth point at the middle third of the left side of the heart forming an imaginary axis (SA) with the second point of this measurement. Upon defining this area, OsiriX gives us a cardiac surface expressed in cm^2^. This measure was based on the hypothesis of the measurements of the long and SA of the cardiac silhouette, which may increase when the ferret develops a heart pathology (Figure 3A).

The axis cardiac area in the ventrodorsal and dorsoventral projections (VD-AREA-AXIS, DV-AREA-AXIS) was created using the OsiriX software axis ROI. For this purpose, four points were placed as if they were the long (LA) and the short (SA) axes of the cardiac silhouette as follows: the first point was placed halfway at the cranial edge of the cardiac silhouette; the second point was placed in the middle third of the heart perpendicular to the imaginary LA of the cardiac silhouette starting from the right side of the heart; the third point was placed at the apex of the heart to form the imaginary axis (LA); and the fourth point was placed at the middle third of the left side of the heart forming an imaginary axis (SA) with the second point of this measurement. As in the previous parameter, OsiriX gave us a cardiac surface in cm^2^ based on the same hypothesis (Figure 3B).

**Figure 4:**
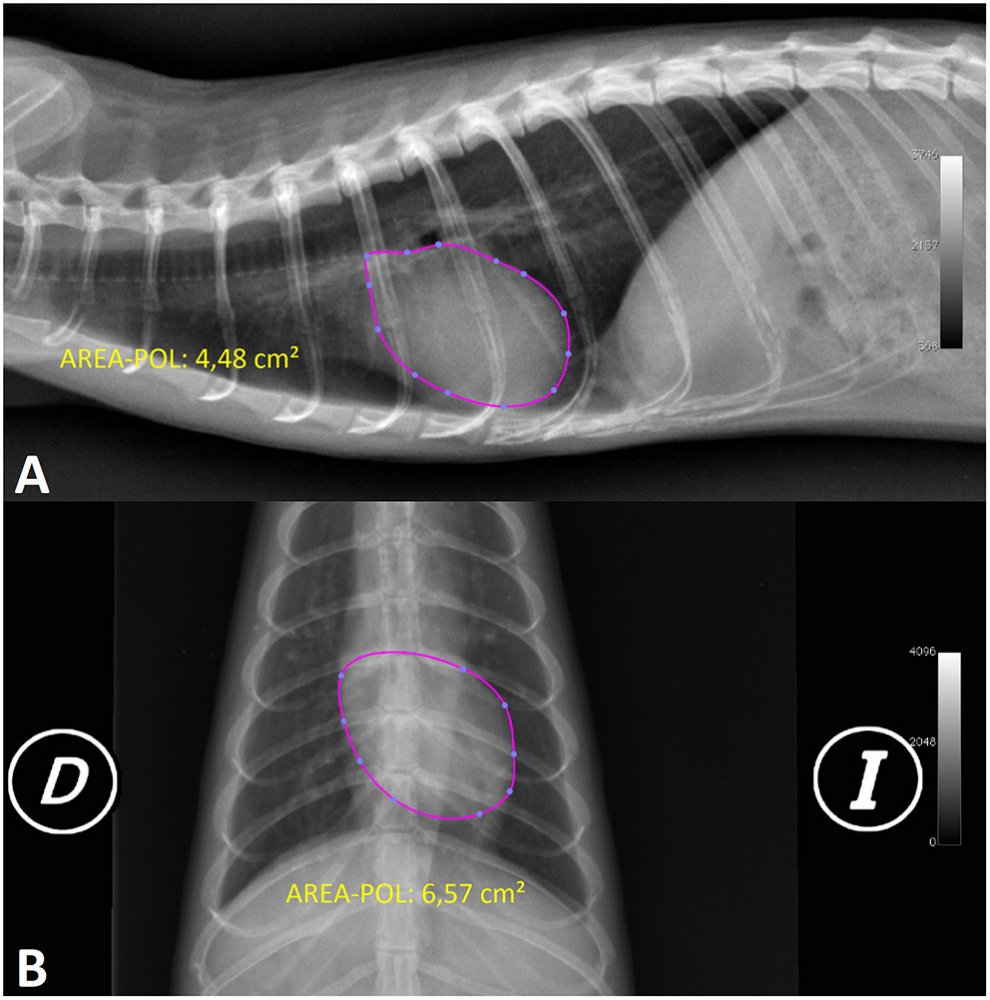
**(A)** X-ray image in the right lateral projection (RL) of the normal thorax of a female ferret and **(B)** ventrodorsal (VD) projection of the normal thorax of a male ferret. The X-rays show a measurement of open polygon cardiac area (AREA-POL) in both projections (D, right; I, left).

The open polygon cardiac area in the right and left lateral projections (RL-AREA-POL, LL-AREA-POL) measurement was made using the open polygon ROI from the OsiriX software, starting along the ventral edge of the tracheal bifurcation and continuing to mark the cardiac silhouette on the left side of the heart until reaching the cranial zone of the descending aorta that contacts the ventral part of the trachea and ending with the junction of this last point with the initial point. If difficulty is encountered in identifying the cardiac silhouette, an alternative method can be followed: upon reaching the area of the visible right cardiac silhouette the next point to be marked should be in the cranial zone of the descending aorta that contacts the ventral part of the trachea (it is easier to visualise this point by looking at the cranial edge of the descending aorta that contacts the dorsal part of the trachea and tracing a perpendicular line to the ventral edge of the trachea). By performing this measurement variation, the ROI automatically curves the cardiac silhouette and can minimise the distortion of the cardiac silhouette in that area. After finishing, OsiriX shows the heart surface value in square centimetres (Figure 4A).

The open polygon cardiac area in the ventrodorsal and dorsoventral projections (VD-AREA-POL, DV-AREA-POL) was created using the OsiriX software open polygon ROI. It was measured by placing nine points as follows: the first point was marked on the left cranial edge at 1 o'clock, followed by three points at approximately the same distance (at 3, 4, and 5 o'clock); the sixth point was placed on the cardiac apex (6 o'clock); and the next four points, which delineated the right cardiac silhouette, were made parallel to the left points (7, 8, 9, and 11 o'clock) before returning to the starting point. By marking the area in this manner, the ROI automatically creates the curves of the cardiac silhouette and can minimise the distortion of the cardiac silhouette in this area (aortic arch, mediastinum). After finishing, OsiriX gives a heart surface value in square centimetres (Figure 4B).

### Statistical Analysis

All variables are expressed as mean, standard deviation, coefficient of variation, and maximum and minimum values. The possible effect of sex on these variables was determined using analysis of variance (ANOVA), with a the *p* < 0.05 accepted as statistically significant. The correlation between different variables was established using Pearson correlation analysis, using the statistical coefficient of determination, *R*^2^, as the quality index of this correlation. ANOVA was used to analyse the projections within the different cardiac scales and the correlation with the projections of the individual measurements. The relationship between different cardiac measurements [sum (L + S); LA] and the anatomical structures used in VHS and SHS scales in the RL projection was also established. To compare cardiac measurements, the correlations of VHS with the different measurements were determined, in addition to their correlation with the sum of the short and LA of the cardiac silhouette in the same projection, by using the same statistical test. Statistical analyses were carried out using SPSS (V23) software licenced to the University of Extremadura.

## Results

Of the 40 ferrets included in the study, 21 were males (52.5%) and 19 were females (47.5%). The mean age of the population was 1.87 ± 0.98 years, with an age range of 0.6–5 years. Males were slightly younger (1.72 ± 0.95 years) than females (2.04 ± 1.04 years). In addition, the age range of females (1–5 years) was larger than that of males (0.6–3.6 years). The mean weight of ferrets was 0.92 ± 0.33 kg with a range of 0.4–1.95 kg, with the males being significantly heavier than females (M: 1.18 ± 0.23 kg; F: 0.64 ± 0.13; *p* < 0.001).

### Radiographic Measurements

The measurements obtained for the different radiographic variables are shown in Table 1. The VHS and SHS measurements showed a lower coefficient of variation than the other variables in the study. Each measurement was compared between the male and female groups (Table 2). The mean values for the RL-VHS, LL-VHS, and DV-VHS were significantly higher in males than females (RL-VHS 5.52 ± 0.28 vs. 5.24 ± 0.2 v, *p* < 0.001; LL-VHS 5.55 ± 0.28 vs. 5.25 ± 0.20 v, *p* < 0.001; and 6.22 ± 0.34 vs. 5.97 ± 0.35 v, *p* < 0.05, respectively). No significant sex differences were found in the measurements in the ventrodorsal projection. In addition, the mean values for RL-SHS and LL-SHS in males were significantly higher than in females (5.10 ± 0.20 vs. 4.67 ± 0.24 s, *p* < 0.001; 5.11 ± 0.20 vs. 4.67 ± 0.28 s, *p* < 0.001). The cardiac area measurements were not only different between sexes but also more homogeneous with decreased coefficient of variation.

**Table 1:** Radiographic measurements for the 40 healthy ferrets.

**Variable**	***n***	**Mean**	**SD**	**CV**	**Min**.	**Max**.
RL-LA (cm)	40	2,91	0,35	12.17	2.32	3.54
LL-LA (cm)	40	2,91	0.34	11.73	2.38	3.60
RL-SA (cm)	40	2.18	0.32	14.58	1.56	2.73
LL-SA (cm)	40	2.20	0.34	15.64	1.51	2.80
VD-LA (cm)	40	3.43	0.46	13.37	2.63	4.29
DV-LA (cm)	40	3.47	0.46	13.34	2.47	4.29
VD-SA (cm)	40	2.27	0.29	12.74	1.55	2.75
DV-SA (cm)	40	2.32	0.34	14.54	1.51	2.97
RL-L + S (cm)	40	5,09	0,65	12,73	3,88	6,20
LL-L + S (cm)	40	5,11	0,66	12,95	3,89	6,40
VD-L + S (cm)	40	5,70	0,71	12,41	4,18	7,03
DV-L + S (cm)	40	5,78	0,76	13,07	4,16	6,97
RL-T4–7 (cm)	40	3.75	0.37	9.81	3.08	4.53
LL-T4–7 (cm)	40	3.75	0.37	9.76	3.06	4.56
VD-T4–7 (cm)	40	3.70	0.37	10.00	2.95	4.48
DV-T4–7 (cm)	40	3.73	0.37	10.03	2.99	4.33
RL-VHS (v)	40	5.39	0.28	5.29	4.80	5.90
LL-VHS (v)	40	5.41	0.29	5.27	4.80	6.00
VD-VHS (v)	40	6.08	0.35	5.69	5.30	6.70
DV-VHS (v)	40	6.10	0.36	5.96	5.30	6.80
RL-S4–7 (cm)	40	4.07	0.40	9.74	3.30	4.87
LL-S4–7 (cm)	40	4.07	0.38	9.40	3.31	4.74
RL-SHS (s)	40	4.90	0.31	6.29	4.00	5.50
LL-SHS (s)	40	4.90	0.33	6.65	4.00	5.50
RL-AREA-AXIS (cm^2^)	40	3.14	0.81	25.67	1.79	4.93
LL-AREA-AXIS (cm^2^)	40	3.17	0.81	25.63	1.89	4.73
VD-AREA-AXIS (cm^2^)	40	3.87	0.94	24.22	2.01	5.56
DV-AREA-AXIS (cm^2^)	40	3.97	1.01	25.30	2.08	5.53
RL-AREA-POL (cm^2^)	40	5.59	1.36	24.35	3.27	8.28
LL-AREA-POL (cm^2^)	40	5.68	1.39	24.44	3.30	8.40
VD-AREA-POL (cm^2^)	40	6.02	1.48	24.58	3.26	8.89
DV-AREA-POL (cm^2^)	40	6.25	1.60	25.62	3.32	9.57

*SD, Standard deviation; CV, Coefficient of variation; Min, Minimum; Max, Maximum; RL, Right lateral projection; LL, Left lateral projection; VD, Ventrodorsal projection; DV, Dorsoventral projection; LA, Long axis of the cardiac silhouette; SA, Short axis of the cardiac silhouette; L + S, Sum of long and short axes of the cardiac silhouette; T4–7, T4 to T7 vertebral body length; VHS, Vertebral heart scale; S4–7, S4 to S7 sternebral body length; SHS, sternebral heart scale; AREA-AXIS, Axis cardiac area; AREA-POL, Open polygon cardiac area*.

**Table 2:** Comparison of radiographic measurements between males and females healthy ferrets.

**Variable**	**Females (*n*)**	**CV**	**Males (*n*)**	**CV**
RL-LA (cm) ^*^	2,58 ± 0,10 (19)	3,96	3,21 ± 0,20 (21)	6,08
LL-LA (cm) ^*^	2,60 ± 0,14 (19)	5,23	3,19 ± 0,20 (21)	6,30
RL-SA (cm) ^*^	1,89 ± 0,15 (19)	8,00	2,44 ± 0,16 (21)	6,60
LL-SA (cm) ^*^	1,89 ± 0,15 (19)	8,05	2,49 ± 0,15 (21)	7,09
VD-LA (cm) ^*^	3,06 ± 0,26 (19)	8,45	3,76 ± 0,33 (21)	8,68
DV-LA (cm) ^*^	3,08 ± 0,29 (19)	9,48	3,81 ± 0,28 (21)	7,24
VD-SA (cm) ^*^	2,05 ± 0,19 (19)	9,16	2,46 ± 0,22 (21)	8.87
DV-SA (cm) ^*^	2,03 ± 0,18 (19)	8,87	2,58 ± 0,20 (21)	7,80
RL-L + S (cm) ^*^	4,48 ± 0,20 (19)	4,51	5,65 ± 0,30 (21)	5,30
LL-L + S (cm) ^*^	4,49 ± 0,22 (19)	4,92	5,67 ± 0,34 (21)	5,99
VD-L + S (cm) ^*^	5,12 ± 0,39 (19)	7,66	6,23 ± 0,48 (21)	7,64
DV-L + S (cm) ^*^	5,11 ± 0,42 (19)	8,28	6,38 ± 0,38 (21)	6,01
RL-T4–7 (cm) ^*^	3,41 ± 0,15 (19)	4,41	4,06 ± 0,20 (21)	4,81
LL-T4–7 (cm) ^*^	3,42 ± 0,17 (19)	4,84	4,05 ± 0,19 (21)	4,78
VD-T4–7 (cm) ^*^	3,36 ± 0,16 (19)	4,84	4,01 ± 0,19 (21)	4,74
DV-T4–7 (cm) ^*^	3,38 ± 0,16 (19)	4,77	4,05 ± 0,17 (21)	4,21
RL-VHS (v) ^*^	5,24 ± 0,20 (19)	4,04	5,52 ± 0,28 (21)	5,11
LL-VHS (v) ^*^	5,25 ± 0,20 (19)	3,88	5,55 ± 0,28 (21)	4,99
VD-VHS (v)	6,01 ± 0,32 (19)	5,28	6,15 ± 0,36 (21)	5,92
DV-VHS (v) ^**^	5,97 ± 0,35 (19)	5,86	6,22 ± 0,34 (21)	5,49
RL-S4–7 (cm) ^*^	3,73 ± 0,25 (19)	6,57	4,37 ± 0,21 (21)	4,88
LL-S4–7 (cm) ^*^	3,75 ± 0,25 (19)	6,62	4,36 ± 0,21 (21)	4,77
RL-SHS (s) ^*^	4,67 ± 0,24 (19)	5,09	5,10 ± 0,20 (21)	3,99
LL-SHS (s) ^*^	4,67 ± 0,28 (19)	6,00	5,11 ± 0,20 (21)	4,01
RL-AREA-AXIS (cm^2^) ^*^	2,39 ± 0,23 (19)	9,43	3,82 ± 0,45 (21)	11,73
LL-AREA-AXIS (cm^2^) ^*^	2,41 ± 0,26 (19)	10,99	3,87 ± 0,41 (21)	10,49
VD-AREA-AXIS (cm^2^) ^*^	3,08 ± 0,45 (19)	14,48	4,59 ± 0,64 (21)	13,86
DV-AREA-AXIS (cm^2^) ^*^	3,06 ± 0,47 (19)	15,28	4,80 ± 0,50 (21)	10,48
RL-AREA-POL (cm^2^) ^*^	4,28 ± 0,30 (19)	7,06	6,78 ± 0,65 (21)	9,63
LL-AREA-POL (cm^2^) ^*^	4,35 ± 0,35 (19)	8,07	6,88 ± 0,68 (21)	9,88
VD-AREA-POL (cm^2^) ^*^	4,72 ± 0,65 (19)	13,77	7,20 ± 0,91 (21)	12,64
DV-AREA-POL (cm^2^) ^*^	4,79 ± 0,66 (19)	13,81	7,57 ± 0,88 (21)	11,63

*CV, Coefficient of variation; RL, Right lateral projection; LL, Left lateral projection; VD, Ventrodorsal projection; DV, Dorsoventral projection; LA, Long axis of the cardiac silhouette; SA, Short axis of the cardiac silhouette; L + S, Sum of long and short axes of the cardiac silhouette; T4–7, T4 to T7 vertebral body length; VHS, Vertebral heart scale; S4–7, S4 to S7 sternebral body length; SHS, sternebral heart scale; AREA-AXIS, Axis cardiac area; AREA-POL, Open polygon cardiac area; values represented by mean ± standard deviation. Statistically significant differences between sexes (*

* *p < 0.001*;

** *p < 0.05)*.

The mean for RL-AREA-AXIS, LL-AREA-AXIS, VD-AREA-AXIS, and DV-AREA-AXIS in males was significantly larger than in females (3.82 ± 0.45 vs. 2.39 ± 0.23 cm^2^, *p* < 0.001; 3.87 ± 0.41 vs. 2.41 ± 0.26 cm^2^, *p* < 0.001; 4.59 ± 0.64 vs. 3.08 ± 0.45 cm^2^, *p* < 0.001; and 4.80 ± 0.50 vs. 3.06 ± 0.47 cm^2^, *p* < 0.001, respectively). In addition, the RL-AREA-POL, LL-AREA-POL, VD-AREA-POL, and DV-AREA-POL were significantly larger in males than in females (6.78 ± 0.65 vs. 4.28 ± 0.30 cm^2^, *p* < 0.001; 6.88 ± 0.68 vs. 4.35 ± 0.35 cm^2^, *p* < 0.001; 7.20 ± 0.91 vs. 4.72 ± 0.65 cm^2^, *p* < 0.001; and 7.57 ± 0.88 vs. 4.79 ± 0.66 cm^2^, *p* < 0.001) (Table 2).

### Correlation Between Cardiac Measurements and Anatomical Structures Used in the Scales

When analysing the correlation between different cardiac measurements and the anatomical structure used in the scales, it was observed that the correlation between the sum of the SA and LA in the right lateral projection (RL-L + S) and the T4–7 vertebral length in the same projection (RL-T4–7) was a strongly positive one, with a coefficient of determination *R*^2^ = 0.83. The correlation between the long axis of the heart, the right lateral projection (RL-LA), and the same vertebral length in the right lateral projection (RL-T4–7) was also positive but with a lower determination coefficient (*R*^2^ = 0.76). However, the coefficient of determination between RL-L + S and RL-LA and the length of the sternebra 4–7 in the same projection (RL-S4–7) was slightly lower with a value of *R*^2^ = 0.75 and *R*^2^ = 0.70, respectively.

### Analysis of Projections

After studying the projection within the variables (VHS and AREA-AXIS), no significant differences were observed either between the lateral projections or between the ventrodorsal and dorsoventral projections. However, significant differences were found between the lateral projections and the ventrodorsal and dorsoventral projections (*p* < 0.05), with the mean value of the ventrodorsal and dorsoventral projections being significantly higher than the lateral projections (*p* < 0.05). The correlation observed between the RL-VHS and the LL-VHS was positive, with a good coefficient of determination (*R*^2^ = 0.77). When the lateral projections were correlated with the ventrodorsal and dorsoventral projections, a poor coefficient of determination was found (between *R*^2^ = 0.21 and *R*^2^ = 0.32), whereas the correlation between VD-VHS and DV-VHS was positive but with a moderate coefficient of determination (*R*^2^ = 0.40). In the AREA-AXIS measurement, the correlation observed between the RL-AREA-AXIS and the LL-AREA-AXIS was positive, with a good coefficient of determination (*R*^2^ = 0.94). When correlating the lateral projections with the ventrodorsal and dorsoventral projections, a good coefficient of determination (between *R*^2^ = 0.80 and *R*^2^ = 0.86) was obtained. Furthermore, the correlation between VD-AREA-AXIS and DV-AREA-AXIS was positive with a very good coefficient of determination (*R*^2^ = 0.89). Regarding the SHS measurements, there were no significant differences between the RL and LL projections (*p* = 0.97). The correlation between the two projections was positive and with a good coefficient of determination (*R*^2^ = 0.84). In the AREA-POL measurement, no significant differences were found between lateral projections, nor between the VD and DV projections. The correlation between the two projections was positive and with a very good coefficient of determination (*R*^2^ = 0.97; *R*^2^ = 0.92, respectively).

### Comparison of Cardiac Measurements

**Figure 5:**
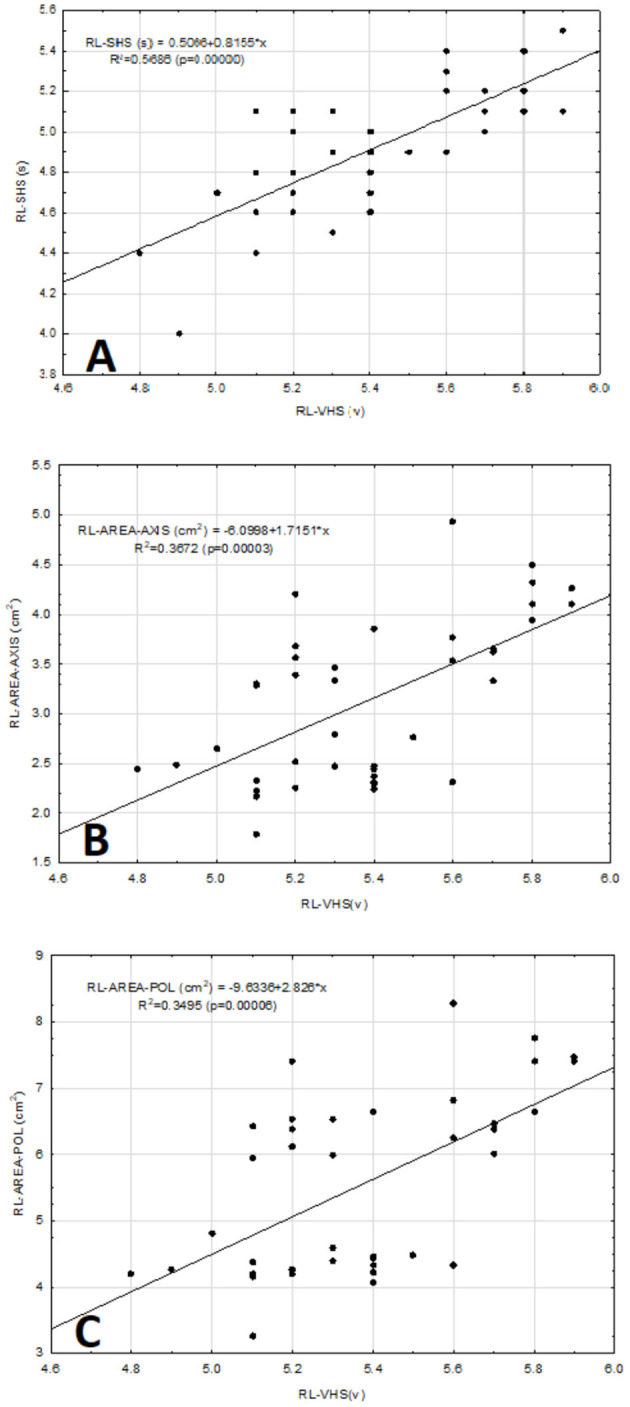
Scatter diagram between vertebral heart scale [VHS (v)] vs. sternebral heart scale [SHS (s)], axis cardiac area [AREA-AXIS (cm^2^)], and open polygon cardiac area [AREA-POL (cm^2^)] in the right lateral projection (RL) obtained from 40 clinically normal ferrets **(A–C)**. The straight line represents the linear equation adjusted to the data points. The corresponding equation is provided in each panel.

The correlation between RL-VHS and the RL-SHS was good, with a coefficient of determination *R*^2^ = 0.57 (Figure 5A). On the contrary, the correlation between the RL-VHS and the cardiac area-axis measurement in the same projection (RL-AREA-AXIS) was weakly positive, with a coefficient of determination R^2^ = 0.37 (Figure 5B). Similarly, the correlation between RL-VHS and the RL-AREA-POL was weakly positive, with a coefficient of determination of *R*^2^ = 0.35 (Figure 5C). However, the trend for both measurements remained the same when compared between sexes.

### Correlation Between Cardiac Measurements and the Measurements Sum of the Long and Short Axes of the Cardiac Silhouette

**Figure 6:**
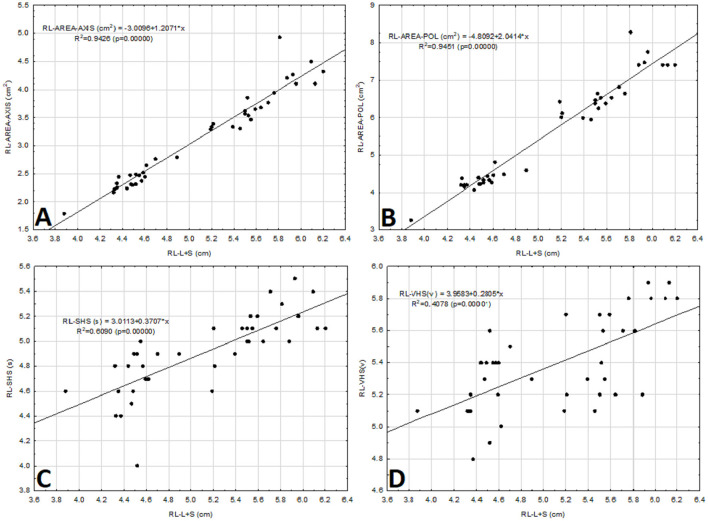
Scatter diagram between the sum of the long and the short axes of the cardiac silhouette [L + S (cm)] vs. the cardiac area-axis [AREA-AXIS (cm^2^)], open polygon cardiac area [AREA-POL (cm^2^)], sternebral heart scale [SHS (s)], and vertebral heart scale [VHS (v)] in the right lateral projection (RL) obtained from 40 clinically normal ferrets **(A–D)**. The straight line represents the linear equation adjusted to the data points. The corresponding equation is provided.

In addition, correlations were made between the different measurements above and the sum of the short and the long axes of the cardiac silhouette. Thus, the correlation between the RL-AREA-AXIS and the RL-AREA-POL with the sum of the long and short axes of cardiac silhouette (RL-L + S) was very strongly positive, with the same coefficient of determination, *R*^2^ = 0.94 (Figures 6A,B). However, the correlation between the RL-SHS and the measurement of the sum of the long and short axes in the same projection (L + S) was moderately positive, with a coefficient of determination of *R*^2^ = 0.61 (Figure 6C). Meanwhile, the correlation between the RL-VHS and (L + S) was weakly positive, with a coefficient of determination, *R*^2^ = 0.41 (Figure 6D).

## Discussion

The minimum age of the animals in this study was taken into account, being the age at which they reach their adult size and sexual maturity (27). Therefore, like other authors (28), we believe that the slight age-related variations were minimised.

When analysing the sexes significant differences in cardiac measurements were found between males and females, in agreement with previous studies (18, 19). However, Vatenburg et al. found no significant differences between male and female ferrets in M mode echocardiographic measurements (26). Based on this background, the authors believe that the sexual dimorphism present in the radiographic cardiac measurements in ferrets may be due to the clear differences in the overall body weight and size between the sexes (13), and they consider it of great importance in this study, as it allowed them to obtain more specific measurements for this species.

The average weight of males was lower than in other studies (18, 19). The authors believe that this is due to the physical characteristics of our European population, which tends to be smaller than the American population (1). Stepien et al. (18) observed a correlation between body weight and cardiac measurements in ferrets. This was verified by Onuma et al. (19), who found statistically significant differences in such measurements in animals weighing <1 kg compared to those ≥1 kg. The possibility of controlling for weight was not considered since the seasonal fluctuation in body weight in this species, with a loss of 40% in summer and an increase in winter, is known (29). This study was carried out along year.

The VHS measured in ferrets in previous studies (18, 19) are modifications of the original study carried out in dogs by Buchanan and Bücheler (3). In this work, a new VHS in ferrets was created following the guidelines of the original Buchanan study in dogs (3), in order to improve the precision of the measurement specifically for this species.

Regarding the SHS, the purpose of this work was to devise another simple method, different from the VHS (3), that compares the size of the heart with a structure of the skeleton, so that the overall size of the body is taken into account. This measurement was conducted in the RL and LL projections, not in the VD and DV projections, due to the overlap of the sternum with the vertebral bodies, which impeded its identification. As in the VHS of this study, significant differences were also found between sexes, with male values being larger than female values in the two projections in which the measurements were made. Therefore, it was considered that to obtain a greater specificity within the measurement, these are the values that must be taken into account.

Regarding the choice of the sternebrae in which the measurement was made, the first, second, and third sternebrae were excluded. The first sternebra due to individual variations in size and shape, as previously reported in other species (3, 4). The second and third sternebrae, due to different sizes and degrees of inclination, due to the thoracic conformation in ferrets. In addition, the measurement was performed by the middle of the sternebral body, because this reduces the separation between the sternebra, and because the costal junction is found ventrally, not allowing the cranial edge to be distinguished from the sternebra. These considerations were taken into account in the choice of the sternebral segment in which the measurement was performed to be important in reducing the variability of this length.

It has been shown that to validate a cardiac scale in which the overall body size is considered, there must be a good correlation between the size of the heart and the anatomical structure with which the proportion is to be established (3, 12, 30). Based on this, a vertebral length scale was selected as an indicator of cardiac size in other species (3, 4). Regarding the VHS measurements, the correlation between the sum of the short and long axes of the cardiac silhouette in the RL-L + S projection and the vertebral length in the same projection (RL-T4–7) was good, and that with the RL-LA it was good but slightly weaker. However, Onuma et al. (19) in their study of VHS measurement in ferrets, used a new segment of vertebral length but did not establish the correlation between heart size and this new segment. Therefore, the authors consider this to be a limitation in their study, as previously Buchanan and Bücheler had established, upon validating their VHS scale in dogs, a very good correlation between (L + S) and the T4-8 vertebral length segment, with a coefficient of correlation R = 0.98 (3). For the feline species, a correlation between (L + S) and the T4-6 vertebral segment was also established in the VHS measurement, with a coefficient of correlation which was good (*R* = 0.78) but less than the canine species (4).

Further, in the current study a good correlation between heart size (RL-L + S, RL-LA) and sternebral length (S4–7) was found in the RL projection; therefore, a sternebral length scale was selected as an indicator of body size. This correlation is lower than that found by other authors in dogs between heart size (RL-L + S) and the length of 3 or 4 sternebrae (*R* = 0.94) (3). However, it is higher than that described by Litster and Buchanan (4) in the feline species, who reported an *R* = 0.67 for the correlation between heart size (RL-LA) and sternebral length (sternebra 2-4). This reaffirms that the correlation determined in this study between heart size and sternebral length is quite good.

There are few studies in the scientific literature that use the sternebrae to obtain different scales of cardiac size, following the study by Buchanan and Bücheler who found a slightly lower correlation in dogs between heart size and sternebral length, compared to the correlation with the vertebral length (3). However, Litster and Buchanan established a measurement in cats according to which the LA of the cardiac silhouette in the lateral projection is approximately the length of three sternebrae measured from S2 to S4 (4). This measurement is limited by using only the LA of the cardiac silhouette. On the contrary, Mostafa and Berry (30) conducted a study in dogs, in which they established a cardiac measurement based on the sternal manubrium (manubrium heart scores or MHS_S_). It is performed by the sum of two ratios: that of the LA of the cardiac silhouette with respect to the manubrium, and the SA of the cardiac silhouette with respect to the manubrium in the RL or VD projections. The correlation between heart size and manubrium was good (30). The problem found in this measurement is mainly the complicated calculation requiring more time to perform. In addition, it could not be performed in all individuals, since manubria that had an abnormal shape or whose cranial margin could not be identified were excluded. On the contrary, the SHS measurement proposed in this work does not have the above limitations and can be used in the assessment of heart size in this and probably in other species.

This study describes new cardiac scales not previously described in the literature, using the OsiriX MD medical imaging software. They are based on determining a cardiac area using the different ROIs in the software. The classical cardiac scales mentioned above for the different animal species are based on those described by Buchanan and Bücheler (3), who sought to standardise the heart size among different canine species by comparing it to a vertebral length, since there was a good correlation between the size of the heart and the vertebral length. However, other authors have argued that the variability of sizes between dogs prohibits the comparison of absolute measurements, but proportions of size to other anatomical structures may be useful in providing a quantitative value (31). This work based in developing these new cardiac scales on what the latter authors described, as there is a marked sexual dimorphism in ferrets. Therefore, the authors believe that the absolute measurements of heart size obtained by the different ROIs can be useful as indicators of heart size in this species. Furthermore, when these measurements were compared between sexes, the coefficient of variation changes from 24–25 % for the mean values in all projections regardless of the measurement, to 7–15% coefficient of variation depending on the projection and the cardiac measurement performed. When comparing the coefficients of variation obtained in this study, with respect to the VHS measure described above, its coefficient of variation between sexes is slightly lower, around 4–6% depending on the projection. Furthermore, in these new cardiac scales, significant differences were found with respect to sex in all the measurements carried out independently of the projection. However, in this work, it is found that the VHS described has significant differences with respect to sex in all its projections except for the VD projection. Therefore, the authors believe that these new cardiac scales are homogeneous, and the values obtained should be used based on sex as a reference.

The AREA-AXIS measurement in all projections was developed from the hypothesis of the measurements of the long and short axes of the cardiac silhouette (3). On lateral X-rays, the cardiac LA reflects the combined size of the left atrium and the left ventricle; the SA includes part of the left and right atria, most likely at the level of the atrioventricular sulcus and atrioventricular valves. In the VD and DV projections, the LA of the heart reflects the combined size of the right atrium and the left ventricle; the SA includes the right ventricle and the left atrium (3, 32). The authors think this cardiac measurement can be somewhat inaccurate because it does not measure the area of all cardiac structures. In addition, several studies have shown that VHS can be used for the diagnosis of different cardiac problems with variable precision (33, 34). However, with the measurement developed in this study, i.e., the AREA-POL, the authors believe that the above-described limitation is resolved since this measurement encompasses all cardiac anatomical structures. The problem encountered in some individuals during the measurement was: first, the difficulty in differentiating the right side of the heart in the lateral projections; secondly, the effect of distortion of the cardiac silhouette in the aortic arch and mediastinum area, when performing the VD and DV projections. These difficulties were minimised upon adjusting the method as described and allowing the ROI to construct the curve of the cardiac silhouette automatically, thus decreasing the effect of distortion of the cardiac silhouette in those areas.

In clinical practise, lateral and DV positioning may be preferred to VD positioning, as they are considered less stressful in cardiac patients (35). Therefore, the authors think it is important to analyse the projections within the different cardiac scales to appreciate the differences that might exist between them. By analysing the projections within the VHS, no significant differences were observed either between the RL and LL projections or between the VD and DV projections. However, it was found significant that the lateral projections differ considerably from the VD and DV projections with the mean value of the VD and DV projections being significantly higher than in the lateral projections. These results are similar to those described above by Stepien et al. (18), who found no significant differences between lateral projections, but noted that the measurements were significantly larger in the VD projection than the lateral projections for their VHS in ferrets. Review of the literature revealed very similar results regarding projections in other species, with no significant differences in VHS between the RL and LL projections (3, 9, 12) or between the VD and DV projections (3, 4, 12). In contrast, some studies in dogs reporting significant differences between lateral projections were found, with VHS being higher in the RL projection compared to the LL (10, 13–15). The possible causes mentioned were the divergence of the X-ray beam and a greater distance of the heart from the cassette (36), or slight changes of the position of the heart within the thorax as a result of gravity when the animal is subjected to different forms of inclination (13). Noticeable differences in VHS between the VD and DV projections of the same dog (3) or within the same race (10) are also described, concluding as a possible cause the fact that in the VD projection there is a magnification produced by the distance between the heart and the X-ray film (3). Regarding the differences between the lateral and VD or DV projections, the authors also found results very similar to those described in this study in dogs, since there have been reports of noticeable differences in VHS in the VD or DV views with respect to the lateral projections. The possible causes include the distance of the heart from the X-ray film, small impact in ferrets, and the fact that the LA in the VD/DV projection includes the right atrium and the left ventricle, whereas in the lateral projections only the left atrium and the left ventricle are included (3).

In addition, in this study, the correlations between the different projections of VHS were calculated, with a strong positive correlation between the RL-VHS and LL-VHS being observed whereas the correlation between the VD and DV projections was positive but moderate. Because of these results, the authors think that the VHS measurement in the RL projection has no advantage over the LL projection. However, although no significant differences in VHS measurements were found in the VD and DV projections, they are variable and behave differently, showing perceptible differences between them to a greater extent than in the lateral projections, giving valuable information.

Regarding the SHS, no significant differences were found in the measurement in the lateral projections, but the correlations obtained between the two projections were slightly better than those of the VHS measurement. Therefore, the authors consider that, like the previous variable, the implementation of the measurement in the RL projection has no advantage over the LL projection.

When analysing the cardiac area measurements (AREA-AXIS and AREA-POL), the same results were found with respect to differences between projections, as for the VHS measurement. However, the correlation between the RL projection and the LL projection was positive but stronger than that obtained in the same projections for the VHS measurement. In addition, the correlation observed between the VD and the DV projections was positive, with a coefficient much higher than the VHS measurement in the same projection. Based on this, the authors believe that, unlike the VHS cardiac measurement, this measurement has no advantages in the implementation of either the RL projection over the LL or the VD projection over the DV.

There is variability between authors regarding the preferred radiographic projection for the performance of cardiac measurements and in particular the most common measurement, i.e., VHS. Buchanan and Bücheler did not show preference between lateral projections but did for the DV projection over the VD projection for cardiac size assessment in dogs, as the contour was more consistent in the DV projection, and because of the magnification found in the VD projection caused by the increase of the distance between the heart and the X-ray film (3). On the contrary, in the feline species, the VD projection is prioritised over the DV one, because the edges of the silhouette of the heart in the DV view are obscured by fat in obese cats (4). In ferrets, cardiac measurements made in the lateral projections are preferred by some authors over those obtained in the VD projection, because the presence of pericardial fat obscures the edges of the cardiac silhouette (18). Considering this background and due to the results obtained in this study, all measurements can be carried out in any of the projections described and only intrinsic factors specific to the animal should be considered to select the most suitable projection at any given moment.

In this study, the authors sought to evaluate what could be the best cardiac measurements for ferrets, trying to evaluate different factors. They found different publications on VHS in other species for the diagnosis of different cardiac problems with variable precision (33, 34). They, therefore, developed a new VHS measurement based on the same guidelines as Buchanan (3) and compared it with the other measurements in this study to determine if they are good indicators of heart problems. However, echocardiography remains the gold standard for diagnosis of cardiac diseases (26).

The correlation between VHS and SHS was good. It is thought to be due to the different anatomical structures used to make the proportion in these scales, which might have a slightly different effect on heart size. Moreover, the trend between the two scales was analysed and determined to be very similar between the two when we stratified for sex. However, a slightly better correlation was found between SHS and (L + S) in the same projection than the one found between that same cardiac measurement and VHS. Therefore, SHS may be a better indicator of heart size than VHS. Based on all this, the authors think that SHS should be considered as an indicator of cardiac size in this species, without, however, being able to establish which is the most accurate. In addition, they believe that this new measurement should be of great use in those individuals who present with vertebral malformations that have been previously described in this species (20).

The correlation of VHS with AREA-AXIS and AREA-POL measurements was the lowest in this study, being weakly positive. These low correlations could be due to the very nature of the measurements as the VHS establishes a proportion between the heart size and an anatomical structure, whereas these new measurements give us an absolute value of the heart size. Therefore, these different measurements of the heart size do not need to correspond. On the other hand, the trend of these two new measurements, when stratified for sex, was very similar to that found in the VHS. In addition, the correlations between AREA-AXIS and AREA-POL measurements with the (L + S) were very strongly positive, whereas the correlation between the (L + S) and VHS, was weakly positive. These trends of association with heart size suggest that these new measurements are good indicators of heart size in this species. Nevertheless, as mentioned in a previous section, VHS has more homogeneous values than the cardiac area measurements, suggesting that it might be somewhat more accurate. However, from a practical point, the cardiac area measurements are preferred, because the VHS calculation is more complicated, therefore, more time-consuming.

This study has several limitations: (1) The effects of the cardiac and respiratory cycle phase may have influenced the results. In ferrets, it has been reported that pericardial fat can obscure the edges of the cardiac silhouette and, therefore, alter the VHS measurement (18). In contrast, the authors believe that these distortions of the cardiac silhouette found on both the right side of the heart and the cardiac apex in the lateral projections, as well as in the mediastinal aortic arch area in the DV or VD projections, are due not only to the presence of pericardiophrenic fat but to the phase of the cardiac cycle since the heart rate in this species is very high. This study did not consider the phase of the cardiac and respiratory cycle. Although this may have slightly influenced cardiac measurements, due to the size and cardiorespiratory frequency of this species, it is very difficult to perform radiographic projection at the appropriate time to minimise this effect. The effect of the phase of the cardiac and respiratory cycle on VHS in dogs has been previously evaluated using fluoroscopy and has been shown to be moderate but not insignificant on the determination of VHS (37). In addition, Doss et al. (9) in a study carried out on chinchillas, mentioned the possibility that both the heartbeats and the movement artefact secondary to breathing may have influenced VHS in this species. (2) The inter- and intra-observer variability of cardiac measurements was not evaluated and is considered an additional limitation of the present study.

In conclusion, this study reports reference intervals of standardised cardiac scales in ferrets obtained in different radiographic projections, as well as of new cardiac measurements made using the OsiriX MD medical imaging software. Due to the great sex dimorphism in this species, the use of the measurements of the cardiac silhouette is recommended when differentiating between sexes, these being more sensitive when assessing the heart size. In addition, all the measurements in this study can be performed in any of the projections described, without significant differences. Therefore, only animal-specific factors should be taken into account to select the most appropriate projection at any given time. Unlike the findings regarding the other measurements of the study, there were no significant differences in VHS, when stratifying for sex, in the VD projection. Therefore, the values should be considered as a reference for the diagnosis of heart disease when adjusting for sex the measurements obtained in the lateral and DV projections. The authors think that the SHS can be used in the assessment of heart size, while another simple method that considers the overall size of the body and can be of use in those individuals who present with vertebral malformations is needed. Although cardiac scales have more homogenous values, the authors recommend the new measurements AREA-AXIS and AREA-POL to evaluate for heart disease as they require less complicated calculations and are less time-consuming. Further studies of sick animals are needed to assess the accuracy of the technique, compared to the VHS method.

## Data Availability Statement

The raw data supporting the conclusions of this article will be made available by the authors, without undue reservation.

## Ethics Statement

Ethical review and approval was not required for the animal study because Ethical review and approval was not required for the animal study because it was a clinical examination that include X-ray study. Written informed consent was obtained from the owners for the participation of their animals in this study.

## Author Contributions

AG, LE, and JJ designed the study, read and approved the final manuscript. AG acquired the data. AG, PR, and LE reviewed the studies, analysed and interpreted the data, and drafted the article. All authors contributed to the article and approved the submitted version.

## Funding

This research was supported by Grant GR21085 to Animal Medicine and Surgery Research Group (CTS041) from Junta de Extremadura.

## Conflict of Interest

The authors declare that the research was conducted in the absence of any commercial or financial relationships that could be construed as a potential conflict of interest.

## Publisher's Note

All claims expressed in this article are solely those of the authors and do not necessarily represent those of their affiliated organizations, or those of the publisher, the editors and the reviewers. Any product that may be evaluated in this article, or claim that may be made by its manufacturer, is not guaranteed or endorsed by the publisher.
